# Formulation of Lipid-Based Tableted Spray-Congealed Microparticles for Sustained Release of Vildagliptin: In Vitro and In Vivo Studies

**DOI:** 10.3390/pharmaceutics13122158

**Published:** 2021-12-15

**Authors:** Khaled H. Al Zahabi, Hind Ben tkhayat, Ehab Abu-Basha, Al Sayed Sallam, Husam M. Younes

**Affiliations:** 1 Tissue Engineering & Nanopharmaceuticals Research Laboratory, Qatar University, Doha P.O. Box 2713, Qatar; k.alzahabi19@imperial.ac.uk (K.H.A.Z.); hind.bt@gmail.com (H.B.t.); 2 Department of Veterinary Basic Medical Sciences, Faculty of Veterinary Medicine, Jordan University of Science and Technology, Irbid 22110, Jordan; abubasha@just.edu.jo; 3 Al-Taqaddom Pharmaceutical Industries, Amman 11947, Jordan; a.sallam@tqpharma.com; 4 Office of Vice President for Research and Graduate Studies, Qatar University, Doha P.O. Box 2713, Qatar

**Keywords:** vildagliptin, lipid excipients, spray congealing, sustained-release, bioavailability study, pharmacokinetics, in vitro–in vivo correlation, type-2 diabetes

## Abstract

Spray-congealing (SPC) technology was utilized to prepare lipid-based microparticles (MP) capable of sustaining the release of Vildagliptin (VG) for use as a once-daily treatment for type 2 diabetes mellitus. VG microparticles were prepared using Compritol^®^ and Gelucire^®^50/13 as lipid carriers in the presence of various amounts of Carbomer 934 NF. The lipid carriers were heated to 10 °C above their melting points, and VG was dispersed in the lipid melt and sprayed through the heated two-fluid nozzle of the spray congealer to prepare the VG-loaded MP (VGMP). The microparticles produced were then compressed into tablets and characterized for their morphological and physicochemical characteristics, content analysis, in vitro dissolution, and in vivo bioavailability studies in mixed-breed dogs. The VGMP were spherical with a yield of 76% of the total amount. VG was found to be in its semicrystalline form, with a drug content of 11.11% per tablet and a percentage drug recovery reaching 98.8%. The in vitro dissolution studies showed that VG was released from the tableted particles in a sustained-release fashion for up to 24 h compared with the immediate-release marketed tablets from which VG was completely released within 30 min. The in vivo pharmacokinetics studies reported a C_max_, T_max_, T_1/2_, and MRT of 118 ng/mL, 3.4 h, 5.27 h, and 9.8 h, respectively, for the SPC formulations, showing a significant difference (*p* < 0.05)) from the pk parameters of the immediate-release marketed drug (147 ng/mL, 1 h, 2.16 h, and 2.8 h, respectively). The area under the peak (AUC) of both the reference and tested formulations was comparable to indicate similar bioavailabilities. The in vitro–in vivo correlation (IVIVC) studies using multiple level C correlations showed a linear correlation between in vivo pharmacokinetics and dissolution parameters. In conclusion, SPC was successfully utilized to prepare a once-daily sustained-release VG oral drug delivery system.

## 1. Introduction

Vildagliptin (VG) is an orally administered selective inhibitor of dipeptidyl peptidase 4 (DPP4) indicated for the treatment of type-2 diabetes [1,2]. It forms a complex with DPP4, resulting in its inhibition and increasing the levels of glucagon-like peptide-1 (GLP-1) and glucose-dependent insulinotropic polypeptide (GIP) hormones. Those hormones are known to maintain glucose homeostasis through the stimulation of the secretion of insulin from the pancreatic islet by increasing the pancreatic β cell mass and inhibiting apoptosis. Consequently, those hormones reduce glucagon levels and suppress the overnight production of hepatic glucose [3].

VG is widely used either alone or with other hypoglycemic agents such as metformin and insulin [4]. It is categorized under the third class of the Biopharmaceutics Classification System (BCS), suggesting a high water solubility and low permeability [5]. VG is rapidly absorbed with a median T_max_ of about 1.5 h after oral dosing with a mean absolute oral bioavailability of 85% [6]. VG is available in the market as an immediate-release (IR) dosage form, with administration requirements of one tablet of 50 mg twice daily to achieve the desired therapeutic effects.

The limitations associated with the marketed VG instantaneous dosage forms offer opportunities for the investigators to design and develop newly controlled and modified delivery systems with a better therapeutic efficacy, reduced administration frequency, fewer side effects, better patient compliance, and subsequently better control over diabetes [7]. Arrays of studies in an attempt to design controlled-release drug delivery of VG have been investigated. VG-loaded MP using different approaches, including wet granulation, solvent evaporation, and ionic gelation methods, have been reported [2,3,8]. Although it offers tremendous advantages over other approaches and technologies, SPC, one of the most current and efficient technologies in preparing lipid-based MP, has never been investigated for developing MP for VG. The SPC can enhance the morphological characteristics of the prepared particles, leading to the preparation of dense, smoothed surface, homogenous, and spherical shape MP [9]. In addition to being rapid and having single-step preparation, the SPC method is an environment-friendly technology. It involves the preparation of MP without using water or organic solvents, which contribute to better dosage form stability [10]. Finally, SPC was reported as a successful approach for preparing modified-release drug formulations, including the antidiabetic drug Glimepiride [10,11,12,13,14,15,16,17].

Compritol^®^888 ATO, which is glyceryl dibehenate, is a hydrophobic mixture of mono- (12–18% *w/w*), di- (45–54% *w/w*), and tri- (28–32% *w/w*) behenate of glycerol with a hydrophilic−lipophilic balance (HLB) of around 2 and a melting point of 70–77 °C. It is well known for its use as a release modifier to retard the release of highly water-soluble drugs [18,19]. Gelucire 50/13, on the other hand, is a stearoyl polyoxyl-32 glycerides nonionic hydrophilic surfactant used in lipid-based oral formulations to enhance the solubility and bioavailability of drugs. It has a melting point of 50 °C and an HLB value of 13 [20,21]. Because of their different HLB values and melting points, SPC can be accomplished without mixing the molten components of both excipients, enabling the preparation of a binary microparticle system controlling the release of water-soluble drugs such as VG.

Therefore, the main aim of our study is to prepare VG-loaded MP based on Gelucire^®^50/13 and Compritol^®^888 ATO lipid matrices using the SPC technique, which will be further pressed into sustained-release tablets, resulting in hydrophobic particles containing VG particles embedded in a continuous hydrophilic bioadhesive matrix. Carbomer 934P NF, also named Carbopol 934P NF, will also be added to the lipid mix to give it a gelling effect with a bioadhesive feature. Carbomer is known to have the ability to adhere strongly to mucosal membranes without causing any irritation. The produced MP will be assessed for the yield, content, thermal, morphological, size properties, in vitro release, and in vivo bioavailability. The pharmacokinetics parameters and in vitro–in vivo correlations (IVIVC) will be constructed using the developed formulations for the marketed Galvus^®^ tablet. 

## 2. Materials and Methods

VG was purchased from Megafine, Mumbai, India. Gelucire^®^50/13 (mp 50 °C) and Compritol^®^888 ATO (mp 70–77 °C) were purchased from Gattefossé, Saint-Priest, France. Carbomer^®^934 NF was purchased from PCCA, Houston, TX, USA. Hydrochloric acid and sodium chloride were purchased from Merck, Darmstadt, Germany. Ammonium dihydrogen phosphate was supplied from BDH, Poole Dorset, England. Potassium orthophosphate, ammonium formate, and formic acid were purchased from Merck, Germany. 

### 2.1. Preparation of VG Microparticles Using Spray Congealing Technology and Tablets Preparation

Compritol^®^ and Gelucire^®^50/13 were heated up to 10 °C above their melting points. VG was dispersed in the molten carrier using a digital high-speed homogenizer (IKA T25, Wilmington, DE, USA) set at 13,000 rpm. VG dispersed in the molten Compritol^®^ was added to the molten Gelucire^®^50/13 drop wisely and under stirring. The molten mix was homogenized, and Carbomer^®^ was added to the mix as per ratios listed in Table 1. The molten mass was pumped into the spray congealer (Buchi B-290, Flawil, Switzerland) using a pump drive (Masterflex, Radnor, PA, USA) through silicone tubing wrapped with silicone heating tape connected to a temperature controller (Cole Parmer digit sense, Vernon Hills, IL, USA). The parameters used for the SPC were 100 °C for the outlet and 7 °C for the inlet temperature with 100% aspiration using the two-fluid nozzle with a diameter of 1.8 mm. The generated MP were collected and stored in desiccators in a cool place until further characterization. The collected MP (F1-F4) were compressed using a single punch laboratory tableting machine (YDP-12, Minhua pharmaceutical Co., Shanghai, China). A mass of MP containing an equivalent of 100 mg of VG was added to the machine’s die and pressed into tablets of 12 mm diameter and 10 mm thickness at a pressure of 10 KN. 

### 2.2. Yield of Production, Drug Recovery, and Particle Size Analysis

The percentage yield of the produced MP using SPC was calculated by dividing the amount of MP of VG produced by the total amount of the drug and lipid carriers initially used. Percentage drug recovery, i.e., % entrapment efficiency was calculated according to Equation (1):

% Drug recovery = (mg of VG in 900 mg sample/100 mg) × 100(1)

The particle size measurement of the MP was determined using Malvern Mastersizer (Malvern, UK). A sufficient amount of the particles were dispersed into the sample cell filled with distilled water. Mastersizer^®^ 2000 Version 5.00 software was used to perform the particle size analysis. The particle size distribution was expressed as the SPAN calculated using Equation (2).

SPAN = *d* (90) − *d* (10)/*d* (50) (2)

where *d*10, *d*50, and *d*90 are the diameters of particles at 10%, 50%, and 90% cumulative volume, respectively.

### 2.3. Differential Scanning Calorimetry (DSC)

The thermal characteristic of the prepared MP was examined using DSC. The analysis was done using a DSC8000 (Perkin Elmer Co., Waltham, MA, USA) equipped with an intra cooling system (IntracoolerII). An amount of 1.5 mg of each of the prepared formulations of the SPC along with the physical mix of carriers and the pure drug were placed into the sampling ban and placed into the DSC under a nitrogen flux (40 mL/min) and heated from 0 to 180 °C at a constant heating rate of 10 °C/min.

### 2.4. X-ray Diffraction (XRD)

The XRD analysis was carried out using D8 Advance (Bruker Co., Karlsruhe, Germany), employing a CuKa radiation source. A 1° divergence slit was used to analyze between the 2θ range 5–35° with a step size of 0.1° and step time of 1 s. All other variables and components were assigned through an auto fitting option in the instrument using the DIFFRAC.EVA software V5.2 (Bruker Co., Karlsruhe, Germany).

### 2.5. Scanning Electron Microscopy (SEM)

The surface and morphological characteristics of the produced MP along with the pure VG were observed using SEM. An amount equivalent to 5–10 mg of the collected MP was spread on a double-sided tape fixed on an aluminum holder, then the sample was spray-coated with a gold film (thickness around 20 nm). Imaging was carried out at 10 kV using Nova NanoSEM^®^ 450 scanning electron microscope (FEI, Hillsboro, OR, USA). 

### 2.6. Dissolution Studies, Release Kinetics, and Aging Effect

Dissolution testing was carried out on tablets of F1-F4 using an Agilent Dissolution Tester (Agilent, Santa Clara, CA, USA). The test was conducted at 37 ± 0.5 °C using USP apparatus I for 24 h in three different dissolution media and time phases mimicking the pH of the GI tract system and following the guidance of the European Medicine Agency of measuring the dissolution in a range of pH between 1.0–7.5 [22]. Initially, the dissolution medium was 500 mL of HCl buffer at a pH of 1.2 for up to two hours. The third and fourth hours of the dissolution were carried out by adding 250 mL of phosphate buffer to the existing buffer medium and adjusting the pH to 4.5. Finally, an amount of 250 mL of phosphate buffer was added to the dissolution medium, and the final pH was adjusted at 6.8 for the rest of the dissolution time. The dissolution tests were performed at 100 rpm. Samples of 10 mL were withdrawn every hour for the first four hours and then every two hours after that. An amount of 10 mL of fresh medium was used to replace the sample withdrawn from the dissolution tester. The samples collected were filtered through a 0.45 µm nylon filter, and the VG released was quantified using a validated HPLC-UV method of analysis [23].

The HPLC system used (Waters^®^ Co., Milford, CT, USA) was equipped with X-bridge Waters^®^ C18 reversed-phase column (150 mm × 4.6 mm; 5 μm). The mobile phase was composed of a mixture of ammonium orthophosphate solution and acetonitrile at a ratio of 85:15 *v/v* and was adjusted to a pH of 6.8 ± 0.3. The flow rate was 1.0 mL/min, and the eluent was monitored at 210 nm. Powdered sample equivalent to 50 mg VG was added to a 50 mL volumetric flask and was dissolved in the mobile phase under sonication for 40 min and then filtered using a 0.45μm nylon filter. Suitable dilution was made until reaching a concentration of 100 µg/mL and 20 µL of the solution was injected into the HPLC system. The drug content was then calculated using a calibration curve of VG standard solutions.

The VG release data were fitted into a range of kinetic models: zero-order, first-order, Higuchi, Hixson–Crowell, and Korsmeyer–Peppas semiempirical models. For each model, both the release rate constants (k) and correlation coefficients (R^2^) were determined by using each of the models’ equations (see Table A2). The release order was considered to follow the model that provided R^2^ values closer to unity [24]. To further assess the release kinetics and construct the IVIVC, T50%, T75%, and T90%, which represent the time taken in hours to achieve 50%, 75%, and 90% in vitro VG release, respectively, were also calculated. The aging effect of the lipid excipients was assessed by conducting dissolution testing on formulations stored at 0 and 6 months at 4 °C, followed by a dissolution profile similarity analysis according to FDA guidelines [25].

### 2.7. Bioavailability Studies 

Five male and four female mixed breed dogs weighing 16 ± 3.1 kg were used to conduct the in vivo bioavailability study on 100 mg tableted F1 and F4 SPC formulations using 50 mg Galvus^®^ immediate-release tablets as a reference drug. The studies were conducted under ethics-approved protocol No. 5/2015 obtained from the Animal Care and Use Committee (ACUC) at Jordan University of Science and Technology in Irbid, Jordan. All dogs were kept for adaptation for two weeks before starting the experiment. The dogs were randomly assigned to three groups and subjected to a crossover study design of three phases with a seven-day washing period. The dogs were fasted 12 h before the dose administration, with free access to water during each period. Food was given after 6 h of the drug administration. A 3 mL blood volume was collected from the forelimb vein at 15 min, 30 min, 1, 1.5, 2, 3, 4, 6, 8, 10, 12, 24, and 48 h from dosing, then centrifuged to separate the plasma. The extracted VG concentrations were quantified using a validated LC/MS method of analysis [23]. 

The area under the plasma concentration−time curve (AUC_0–t_ and AUC_0–__∞_) was calculated using the linear trapezoid method. The elimination rate constant (K_e_) was determined using the least-square regression analysis of terminal log-linear portions of the plasma concentration profile. The maximum concentration (C_max_) and the corresponding peak time (T_max_) were determined by the inspection of the individual drug plasma concentration−time profiles. The slope of the terminal phase of the semi-log time–concentration curve was determined by linear regression and was converted to an elimination half-life (T_1/2_) by multiplying the reciprocal by 0.693. Mean residence time (MRT) = AUMC/AUC, where AUMC is the area under the moment curve. The relative bioavailability (F) of the test formulation was calculated as follows: F = AUC_0–t (test)_/AUC_0–t (reference)_ × 100%. 

### 2.8. Pharmacokinetic and Statistical Analysis

Phoenix WinNonlin^®^ 7.0 (Pharsight, Menlo Park, CA, USA) was used to calculate the pharmacokinetic parameters. SPSS^®^ 22 (SPSS Institute Inc., Chicago, IL, USA) was used to conduct ANOVA to compare the PK parameters between the tested and reference formulations. The t-test was used to compare the SPAN values between the F1 and F4, where *p* < 0.05. Linear regression analysis was performed to assess the in vitro–in vivo correlation. 

### 2.9. In Vitro–In Vivo Correlation (IVIVC) Studies

Multiple C level correlation was used to construct a relation between in vitro dissolution data and in vivo PK parameters using linear regression. The Weibull model was used to calculate the dissolution parameters to build up such an IVIVC model by plotting those parameters against C_max_, AUC, and T_max_ using the Sigma Plot software. The Weibull model is described using Equation (3) [26].

(3) $$M_{t}=M_{0}\;\left[1-e^{-\left(\;\frac{\;{\left(t-\tau \right)}^{b}}{a}\right)}\right]$$

where *M_t_* is the amount of drug dissolved at time *t*, *M*_0_ is the total amount of the released drug, *τ* is the lag time for release determined from the dissolution results, parameter a delineates the time dependence, and parameter *b* defines the shape of the dissolution curve progression (*b* = 1 exponential; *b* > 1 sigmoid; and *b* < 1 parabolic).

## 3. Results and Discussion

### 3.1. Yield, Content, and Particle Size Analysis 

The yield of the produced spray-congealed MP exceeded 71% of the initial amount introduced to the spray congealer for all of the prepared formulations. Table 2 reports the analysis outcome related to the SPC percentage production yield of MP and the percentage VG recovery in the prepared formulations. The loss in MP production yield observed was due to the adherence of the lipid carriers to the wall of the congealing chamber. Other variables, including the atomization temperature, air pressure, and the physicochemical properties of the API and lipid materials used in the experiment, were also reported to impact the yield of the SPC process [27]. The reported average process yield in the literature for MP prepared using SPC was in the range of 70%, which is similar to what was obtained for our formulations, as reported in Table 2 [27,28]. The drug recovery analysis revealed that VG was within the accepted USP compendial range of 90–110% (Table 2). The small variations observed could be attributed to the loss of the lipid carrier in the SPC machine during the fabrication process, which could have affected the ratio between the drug and the lipid carriers. Such possible discrepancies between the analyzed content of the drug in the MP and the theoretical values were previously reported in a study aimed to utilize SPC in order to enhance the bioavailability of the drug extract [29].

F1 and F4 formulations were then chosen as representatives of the prepared MP formulations. Therefore, they were subjected to further in vivo and in vitro testing because they sat at the far ends of the dissolution testing profiles (see dissolution data), so they fulfilled the IVIVC requirements. In addition, F1 contained no Carbomer in its composition, while F4 contained Carbomer’s highest amount, so further testing was needed to investigate the impact of Carbomer’s inclusion on the formulation. 

**Figure 1:**
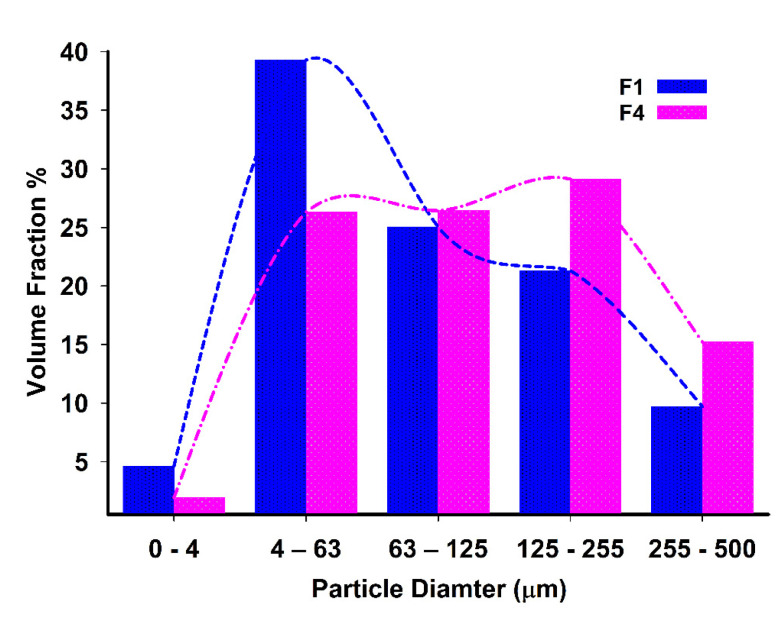
Particle size distribution for F1 and F4 determined using Mastersizer^®^.

Figure 1 shows the results of particles size and distribution analysis carried out on F1 and F4 (See Table A1 for further details). A negatively skewed distribution for F1 and F4 was observed. The median particle size for F1 was around 74 μm, while that for F4 was 112.4 μm. Almost 15.2% of F4 had the particle size of 255–500 μm, while only 9.7% of F1 had the latter size. Data also show that F4 possessed a larger particle size fraction than F1, which could be attributed to the significant contribution of the added Carbomer^®^ on increasing the particle size. On the other hand, the span values for F1 and F4 were 2.52 and 3.21, respectively. These results indicated that F4 has a statistically (*p* < 0.05) wider size distribution and higher polydispersity as well.

### 3.2. DSC and XRD Analysis

**Figure 2:**
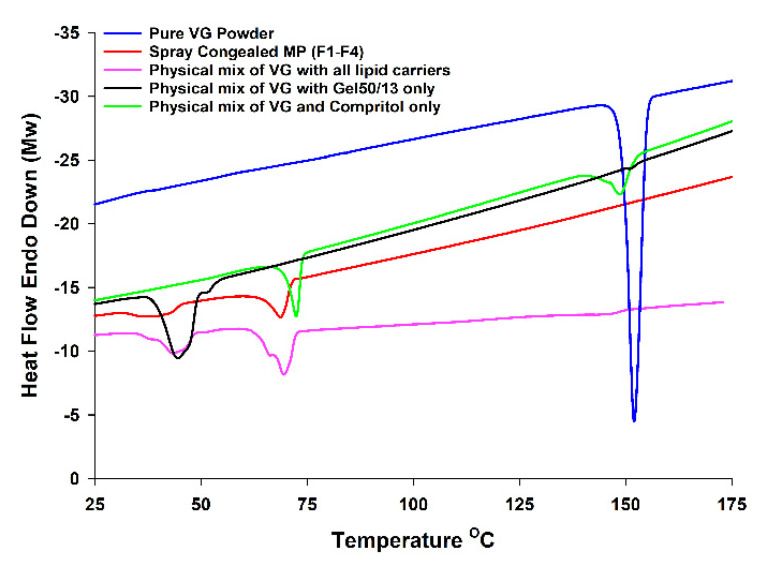
DSC thermograms of pure VG and various formulations.

As shown in Figure 2, an endothermic peak at 150 °C corresponding to the melting point of pure VG was observed. Gelucire^®^50/13 and Compritol^®^ showed endothermic peaks corresponding to their melting points at 50 °C and 72 °C, respectively. A physical mixture of the VG and Gelucire^®^50/13 (1:8) showed a very small endothermic peak at 150 °C, which corresponds to VG, while the same ratio of the drug mixed with the Compritol^®^ showed a larger peak of VG as an indication that VG was better solubilized in the molten Gelucire^®^50/13 when compared with Compritol. On the other hand, the spray congealed MP representing all of the formulations (Table 1) showed only the endothermic peaks, which correspond to the lipid carriers but not VG. The DSC results indicate that VG was dispersed in its semicrystalline form, mostly in the Gelucire^®^50/13 fraction of the mixture.

**Figure 3:**
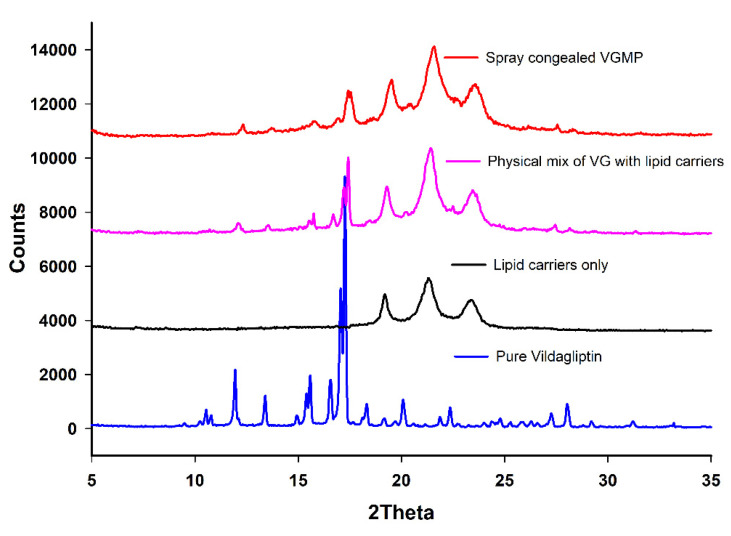
X-ray diffraction patterns of lipid carriers, and pure and formulated VG in F4.

As shown in Figure 3, the X-ray diffraction analysis of VG powder showed several peaks between Bragg angle 2θ of 10 to 27, with a high intense characteristic peak at 17θ. The mix of lipid carriers showed three different peaks in the range between 18 and 25θ. The XRD crystallography of VG in the lipid carriers, particularly those of SPC formulation, showed that much of VG peaks disappeared or decreased intensity compared to pure VG or VG in other mixes, confirming that a significant portion of the loaded VG was dispersed in its semicrystalline to amorphous form in the molten lipids. The XRD results of the lipid carriers matched those reported earlier [9].

### 3.3. Morphological Analysis

**Figure 4:**
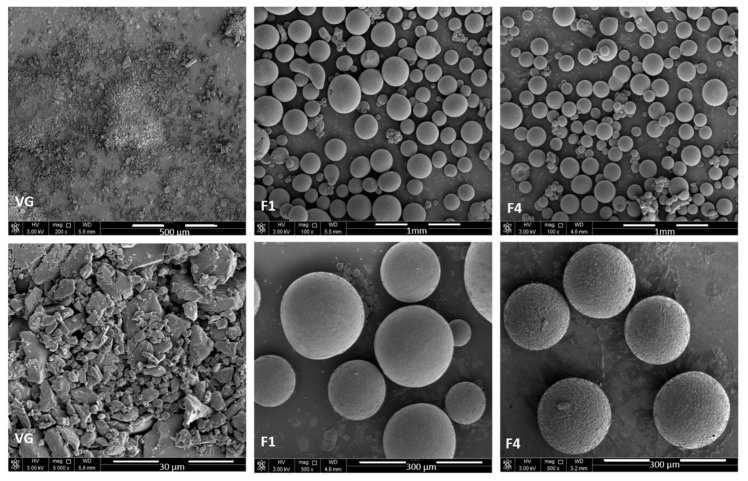
SEM images of VG crystalline powder (using 200× and 5000× magnification), F1, and F4 microparticles (using 100× and 500× magnification).

As shown in Figure 4, the VG powder possessed a crystal-like shape. In contrast, the F1 microparticles showed a dense spherical shape with a smooth surface, similar to what was observed previously in other reported studies involving Gelucire^®^50/13 or Compritol^®^ as lipid excipients [29,30]. On the other hand, the VG microparticles containing Carbomer^®^ also tended to form spherical microparticles, but with more rough surfaces, suggesting that Carbomer^®^ inclusion had a role in increasing the size and roughness of the surface of the prepared SPC particles. 

### 3.4. Dissolution Studies, Aging and Release Kinetics

**Figure 5:**
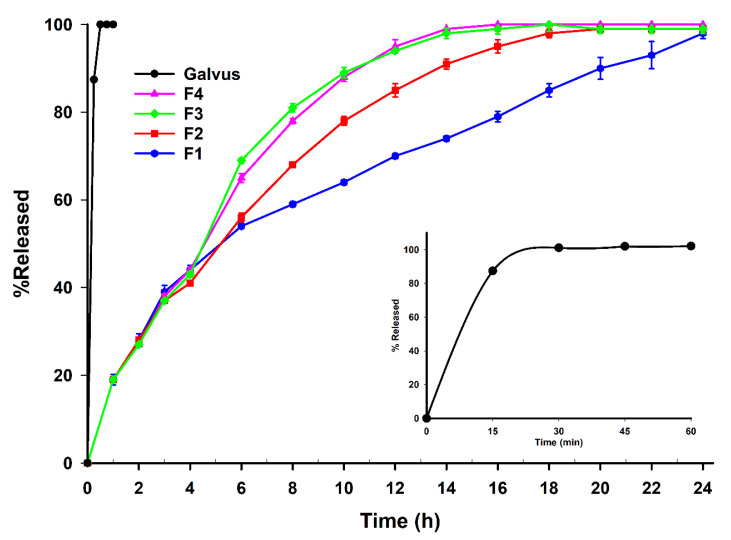
Dissolution profiles for the prepared formulas, as per Table 1.

The in vitro dissolution testing reported in Figure 5 shows a rapid and complete release of VG from the marketed reference tablets, Galvus^®^, with 100% release achieved within 30 min. On the other hand, VG was released from all of the prepared tablet formulations in a sustained-release manner, with F1 showing the slowest release profile compared with the rest, suggesting that Carbomer^®^ inclusion increased the release rate of VG by increasing the ratio of the hydrophilic components in the formulations. Carbomer^®^ is a water-soluble excipient that is reported to have a role in the dissolution enhancement of many drugs [31]. The presence of Carbomer^®^ in the other formulations also resulted in a bi-modal release profile of VG, caused by its gelling effect. Carbomer is known not to dissolve, but swells to a remarkable extent in water after neutralization to form a gel, further contributing to the sustained-release effect on VG [32]. In addition, although F4 was formulated using VGMP of a higher SPAN and larger size than F1, their effect on the release was minimal due to the pressing of MP into tablets. Therefore, excipients’ hydrophilicity/hydrophobicity and gelling characteristics predominated the effect on the release of VG from pressed tablets. The release of the VG from the formulations prepared by SPC using the lipid carriers showed better results when compared with the direct compression of VG with different other release polymers, as the complete release was achieved within 10 h compared with 18–24 h for the SPC formulas [33]. 

**Figure 6:**
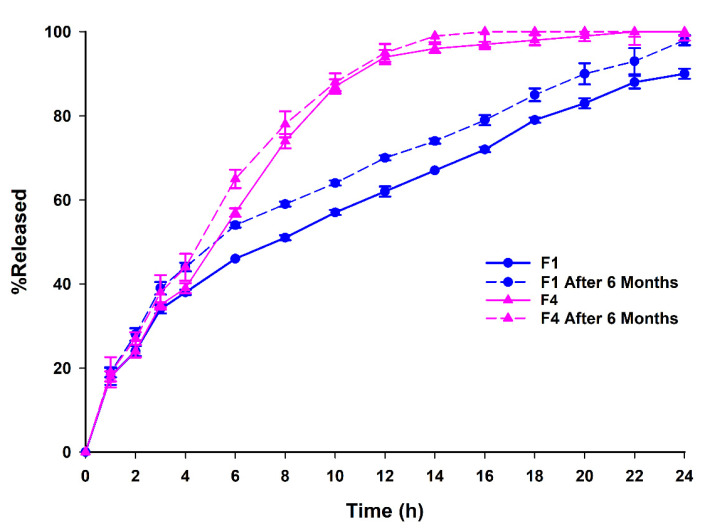
Dissolution profiles of F1, F4 at day 1 and after 6 months of storage at 4 °C.

F1 and F4 were also subjected to aging effect investigation, as many studies have reported on the polymorphism of Gelucire^®^50/13, which led to changes in the release profile of many drugs [34]. As shown in Figure 6, the release profiles for VG were not altered during the storage period of 6 months at 4 °C. On the other hand, the calculated similarity factors (f2) for F1 and F4 were found to be 60 and 76, respectively. This confirmed that the dissolution profiles of the stored formulations were similar to those of the freshly prepared formulations. The aging effect did not significantly affect the drug release.

As mentioned earlier, the release data were fitted into a range of kinetic models, including zero-order, first-order, Higuchi, Hixson–Crowell, and Korsmeyer–Peppas semiempirical models (Table A2) [35]. The constants and coefficient of determinations (R^2^) for each model are reported in Table A3. The Higuchi kinetic model is a simple model that is frequently used in swellable polymer delivery systems. The exponent (*n*) distinguishes the Korsmeyer–Peppas kinetic model from the Higuchi kinetic model; hence the Higuchi model’s application is limited compared with the Korsmeyer–Peppas kinetics model. The Fickian diffusion, anomalous transport, and Case II transport are all included in the Korsmeyer–Peppas kinetics model, which takes into consideration the various release kinetics depending on the value of (*n*). As a result, the Korsmeyer–Peppas kinetic model is frequently used in matrix drug delivery systems, where the processes of sustained release are frequently complex, such as diffusion, swelling, and erosion [36].

As can be seen from the analysis in Table A3 and the dissolution profiles in Figure 5, the initial 50% release in the first 6 h of F1 followed a typical non-Fickian diffusion. After that and up to 24 h, the release of F1 followed typical zero-order kinetics (R^2^ = 0.997), where the dissolution of VG was only a function of time. It was previously reported that many other drugs, such as Theophylline, followed the Higuchi diffusion mechanism once prepared by SPC using Compritol^®^ alone [14]. However, in our case, all the other formulations (F2–F4) followed the Korsmeyer–Peppas model with anomalous transport, which indicated that the release of the drug depended on combined swelling and diffusion mechanisms of release [37]. This was clearly demonstrated through the bimodal release pattern that the carbomer inclusion in the formulation caused. Finally, Galvus^®^ marketed tablets followed a typical first-order release profile in which the release rate was proportional to the concentration of VG, as reported earlier [38].

### 3.5. In Vivo Bioavailability Studies

**Figure 7:**
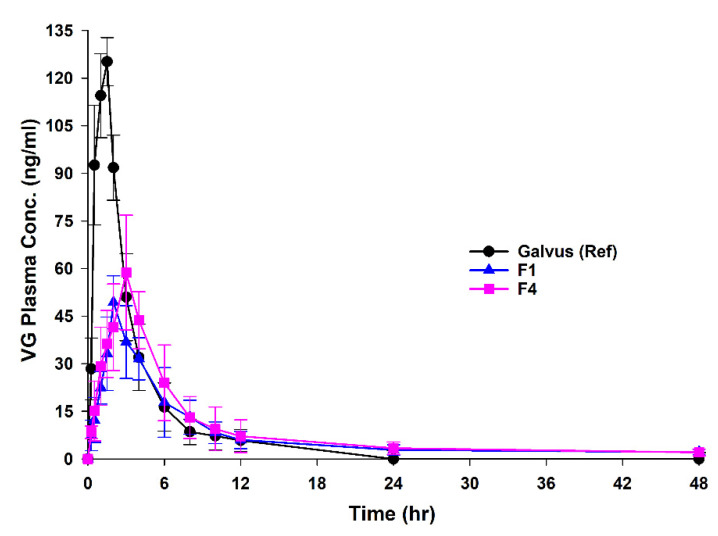
Plasma concentration-time profile of 100 mg F1 and F4 tested tablet formulations versus Galvus^®^ 50 mg as a single oral dose of VG. Values are mean ± SD (*n* = 9).

**Figure A1:**
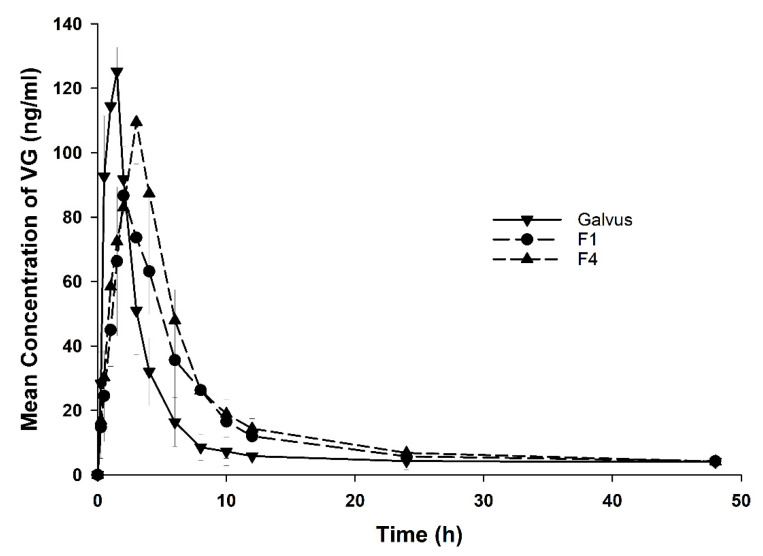
Time versus plasma concentration profiles of F1 and F4 tested formulations versus Galvus^®^ reference (before normalization).

The analysis of the pharmacokinetic parameters was done after the dose normalization process due to the discrepancies in the administered dose between the reference formulas (50 mg) and the tested ones (100 mg). Figure 7 reports the in vivo release of F1, F4, and reference Galvus^®^ after the normalization process (See Figure A1 for data before normalization). As such, VG plasma concentrations were divided by 2 to compare the final pharmacokinetics parameters, knowing that the pharmacokinetics parameters of VG are linear in the range of dose from 25–200 mg [39]. Table 3 summarizes the pharmacokinetic parameters, which reports a T_max_ of 1.05 h for Galvus^®^ that was significantly lower than the T_max_ of F1 and F4. The C_max_ of Galvus^®^ was significantly higher than those of F1 and F4, while the mean residence time (MRT) and T_1/2_ were significantly higher for F1 and F4 compared to the Galvus^®^. The elimination rate K_e_ for F1 and F4 was significantly lower when compared to the marketed Galvus^®^. The AUCs were comparable for the three formulations after dose normalization, indicating that the bioavailability of the tested formulations and the reference were similar and not compromised upon designing the sustained-release dosage form. Recent comparative bioequivalence study in human subjects using 50 mg Galvus^®^ as a reference reported that VG had a T_max_ of 1.79 ± 0.27 h with a half-life of elimination of approximately 2.63 h [40].

### 3.6. In Vivo–In Vitro Correlation (IVIVC)

IVIVC is usually developed to enable the use of in vitro dissolution testing as a surrogate for bioavailability studies. Regulatory authorities usually recommend IVIVC model development for most modified release dosage forms. The main advantage of IVIVC is that it provides a mechanism for evaluating the change in the in vivo absorption based on in vitro dissolution changes when there are small modifications in a formulation. In our studies, the discrepancies in the release and absorption rates between the reference drug, F1 and F4, fulfill one of the fundamental considerations for establishing successful IVIVC relationships [41]. This type of correlation is likely to exist in our case, as VG has a high solubility, and its dissolution is the rate-limiting factor in the process of drug absorption, as demonstrated in the conducted in vivo studies. From the four levels of correlation, level A is considered the most powerful and reliable. However, as we were comparing our sustained-release formulations to an immediate-release reference, it was impossible to construct a point-to-point comparison to establish a level A correlation throughout the whole release profile. As such, we attempted to establish multiple level C correlations. 

At this level of correlation, pharmacokinetics parameters, including C_max_, AUC, and T_max_, are correlated with different dissolution time levels covering the early, middle, and late stages of the dissolution profiles [42]. The C_max_, T_max_, and different partial AUCs were plotted against the corresponding in vitro data obtained through the Weibull model (see Equation (3)), as reported in Table 4 [43].

**Figure 8:**
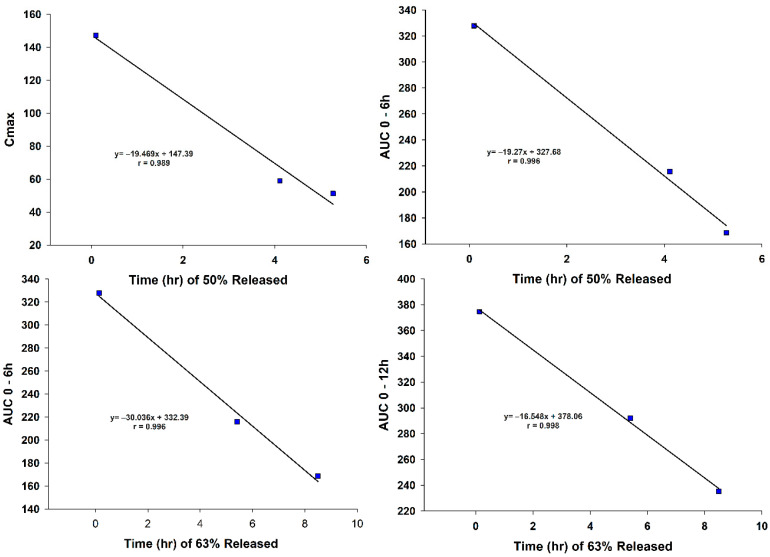
IVIVC—C_max_ and AUC versus time.

The C_max_ of VG in the plasma showed a linear relationship with T_50%_. i.e., the 50% releasing time of the VG. The correlation coefficient of r = 0.989 demonstrated the linear correlation between the C_max_ and T_50%,_ as reported in Figure 8. 

**Figure 9:**
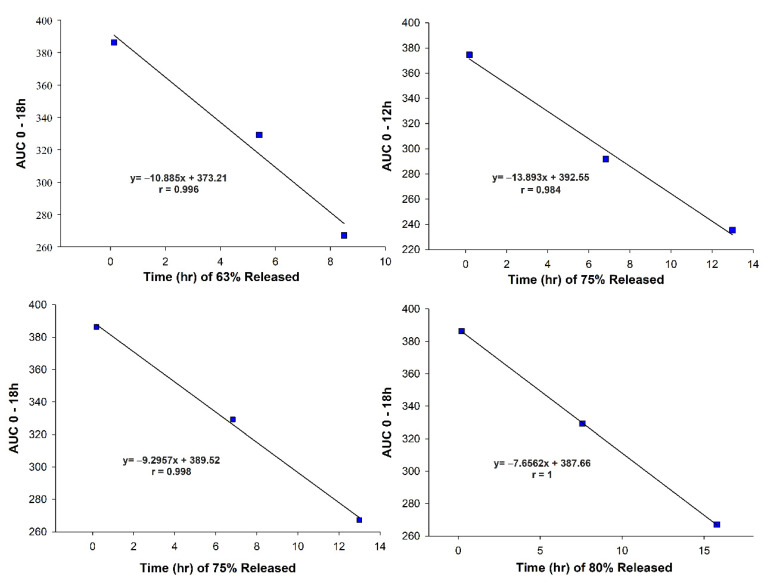
IVIVC—AUC versus Time.

There was a linear correlation between the extent of absorption and different parameters of in vitro release. The AUC_0–6_ was correlated to the T_50%_ and T_d_ with a correlation coefficient of r = 0.996, confirming a linear relationship between the studied parameters, as shown in Figure 8. Furthermore, the AUC_0–12_ was correlated with T_d_ and T_75%_ with a correlation coefficient of r = 0.998 and r = 0.984, respectively, but the higher correlations were with the Td, as shown in Figure 8 and Figure 9. There was a linear relationship between the AUC_0–18h_ and the time for releasing 80% of VG (T_80%_) with a correlation coefficient reaching unity. Similar results were obtained with T_75%_ and T_d_ with a correlation coefficient of r = 0.998 and r = 0.984, respectively. These results showed a linear relationship between the extent of absorption of the VG for different times with the corresponding in vitro dissolution results, as demonstrated in Figure 8 and Figure 9.

## 4. Conclusions

SPC was successfully utilized to prepare solid lipid MP loaded with VG. The produced microparticles were spherical with a high yield of production. The thermal analysis showed that the VG melting endotherm disappeared because it dissolved in the melted Gelucire^®^50/13, while it did not dissolve in Compritol. The in vitro and in vivo analysis showed that the VG was released in an extended-release profile compared to the immediate release marketed drug.

The pharmacokinetic parameters for the three different formulations (Galvus^®^, F1, and F4) showed no significant differences for the extent of absorption. However, the T_max_, C_max_, MRT, and T_1/2_ were significantly different between the SPC and the reference formulation, indicating that VG was released in a sustained-release fashion compared to the marketed drug. The IVIVC results showed that multiple level C correlation had been established, and various pharmacokinetic parameters were found to have a linear relationship with the dissolution parameters, including C_max_ and AUC. However, such a level of correlation and the fact that only three formulations were utilized in building up those linear relationships can only be used as a guide in formulations’ development, and not as a conclusive biowaiver or alternative to conducting bioavailability studies. Constructing level A correlation built on point-to-point comparison between in vitro and in vivo data is a future plan for our group once a sustained-release reference of VG is available. The presented results could constitute a promising approach and guide for developing new pharmaceuticals, while reducing the need for in vivo studies during the formulation and development stage of drugs similar to VG.

## Figures and Tables

**Table 1:** The prepared VG formulations and their composition.

Formula Code	VG (mg) *	Compritol^®^ (mg)	Gelucire^®^ 50/13 (mg)	Carbomer^®^ (mg)
F1	100	500	300	0
F2	100	500	280	20
F3	100	500	260	40
F4	100	500	210	90

* The 100 mg VG corresponds to 11.11%, representing VG’s theoretical content in each formula.

**Table 2:** Yield and VG content analysis of the prepared formulations.

Formula	% Yield of MP	% VG Recovery
F1	79.00	98.80
F2	71.50	95.40
F3	73.00	90.10
F4	76.00	94.40

**Table 3:** Summary of normalized pharmacokinetic parameters of the 100 mg F1 and F4 tested tablet formulations versus Galvus^®^ 50 mg as a single oral dose of VG. Values are mean ± SD (*n* = 9).

Formula	C_max_ (ng/mL)	T_max_ (h)	Ke (1/h)	T_1/2_ (h)	MRT (h)	AUC_0–48_ (h·ng/mL)	AUC_0–∞_ (h·ng/mL)	Rel. Bioav.
Galvus^®^	147.3 ± 61.3	1.06 ± 0.58	0.34 ± 0.09	2.16 ± 0.56	2.87 ± 0.62	372.5 ± 50.6	388 ± 50.5	1.0
F1	51.19 ± 11.9 *	2.66 ± 0.86 *	0.09 ± 0.03 *	8.816 ± 2.9 *	9.27 ± 3.66 *	330 ± 99.1	359 ± 113	0.87
F4	59 ± 14.7 *^,#^	3.42 ± 1.1 *^,#^	0.07 ± 0.0 *^,#^	10.54 ± 2.32 *	9.86 ± 21 *^,#^	412.4 ± 108.1	443 ± 120.7	1.1

* Significant difference from reference formulation (*p* < 0.05), ^#^ significant difference from F1 formulation (*p* < 0.05).

**Table 4:** The parameters of the Weibull release modeling.

Parameters of Weibull Model	Galvus^®^	F1	F4
R^2^	0.999	0.989	0.990
Time for 25% release (T_25%)_ (h)	0.045	1.674	1.956
Time for 50% release (T_50%)_ (h)	0.098	5.272	4.111
Time for 63% release (T_d_) (h)	0.135	8.495	5.406
Time for 75% release (T_75%_) (h)	0.180	12.993	6.827
Time for 80% release (T_80%)_ (h)	0.205	15.776	7.575

## Data Availability

Not applicable.

## Appendix A

**Table A1:** Particle size analysis summary for F1 and F4.

Formula	Particle Size Volume Percentage of Size Range (μm)					SPAN
	0–4	4–63	63–125	125–255	255–500	
F1	4.63	39.29	25.04	21.32	9.70	2.516 *
F4	1.93	26.30	26.44	29.12	15.21	3.205 *

* Significance *p*-value < 0.05.

**Table A2:** A list of empirical and semiempirical release models used to access VG release from the reference and tested formulations.

Equation #	Model	Equation *	Plot
1s	Zero-Order	$M_{t}=M_{0}-k_{0}t$	The amount of released drug versus time
2s	First-Order	$M_{t}=M_{0}e^{-kt}$	The decimal logarithm of released bioactive versus time would yield a straight line with a slope of −K/2.303
3s	Higuchi	$M_{t}=K_{H}\sqrt{t}$	Percentage of released drug versus square root of time
4s	Hixson-Crowell	$\sqrt{M_{t}}=kt+\sqrt{M_{0}}$	The cubic root of remaining drug in matrix/tablet versus time
5s	Korsmeyer-Peppas	MtM∞=ktn	Log percentage of released bioactive versus log time

* Where *M_t_* is the amount of drug released at time *t*, *M*_0_ is the initial amount of *M*, and *K* is the release/dissolution rate constant. The *n* value in the Korsmeyer Peppas equation is the release exponent used to characterize different releases for cylindrical-shaped matrices like tablets.

**Table A3:** Parameters and determination coefficients of the linearization of VG release from Galvus and prepared formulations.

Model	Formulations														
	F1			F2			F3			F4			Galvus^®^		
	R^2^	K	*n*	R^2^	K	*n*	R^2^	K	*n*	R^2^	K	*n*	R^2^	K	*n*
Zero order (0–6 h)	0.912	3.448	-	0.87	3.9	-	0.782	3.867	-	0.851	4.29	-	0.104	0.91	-
Zero-order (post 6 h)	0.997	2.445		-	-	-	-	-	-	-	-	-	-	-	-
First order	0.964	0.117	-	0.921	0.15	-	0.944	0.18	-	0.967	0.19	-	0.986	8.53	-
Higuchi model	0.994	20.19	-	0.974	22.52	-	0.921	23.69	-	0.962	24.66	-	-	-	-
Hixson Crowell	0.929	0.03	-	0.920	0.04	-	0.945	0.05	-	0.960	0.05	-	-	-	-
Korsmeyer Peppas	0.990	22.08	0.47	0.987	23.76	0.48	0.965	28.16	0.43	0.981	25.97	0.48	-	-	-
